# Hypoxia and interleukin-1-primed mesenchymal stem/stromal cells as novel therapy for stroke

**DOI:** 10.1007/s13577-023-00997-1

**Published:** 2023-11-21

**Authors:** Maryam Adenike Salaudeen, Stuart Allan, Emmanuel Pinteaux

**Affiliations:** 1 Faculty of Biology, Medicine, and Health, Division of Neuroscience, University of Manchester, Manchester, UK; 2 Department of Pharmacology and Therapeutics, Ahmadu Bello University, Zaria, Nigeria

**Keywords:** Priming, Mesenchymal stem/stromal cell, Stroke, Ischemia, Hypoxia

## Abstract

Promising preclinical stroke research has not yielded meaningful and significant success in clinical trials. This lack of success has prompted the need for refinement of preclinical studies with the intent to optimize the chances of clinical success. Regenerative medicine, especially using mesenchymal stem/stromal cells (MSCs), has gained popularity in the last decade for treating many disorders, including central nervous system (CNS), such as stroke. In addition to less stringent ethical constraints, the ample availability of MSCs also makes them an attractive alternative to totipotent and other pluripotent stem cells. The ability of MSCs to differentiate into neurons and other brain parenchymal and immune cells makes them a promising therapy for stroke. However, these cells also have some drawbacks that, if not addressed, will render MSCs unfit for treating ischaemic stroke. In this review, we highlighted the molecular and cellular changes that occur following an ischaemic stroke (IS) incidence and discussed the physiological properties of MSCs suitable for tackling these changes. We also went further to discuss the major drawbacks of utilizing MSCs in IS and how adequate priming using both hypoxia and interleukin-1 can optimize the beneficial properties of MSCs while eliminating these drawbacks.

## Introduction

Stroke is one of the leading causes of death worldwide and the major cause of long-term adult disability [1]. Ischaemic stroke (IS) accounts for more than 80% of all stroke incidents [2]. During IS, blood vessels are obstructed by a thrombus or embolus, thus reducing, or stopping the blood supply to the affected brain region. Consequently, the affected region suffers from low oxygen and energy supply and becomes infarcted with a surrounding penumbra of salvageable cells. It takes about 2 weeks for new blood vessels to form [3] and replace some of the blocked and destroyed blood vessels. Before this process is complete, a wide array of disabilities and loss of function would have occurred, and since vessel occlusion occurs predominantly in the middle cerebral artery that supplies parts of the frontal, parietal, and temporal brain lobes with nutrient and oxygen, [4], functions of this brain regions including memory, motor coordination, and gait are affected and constitute the commonest symptoms of ischemic stroke such as hemiparesis, hemiplegia, aphasia, and dysarthria. In addition to thrombectomy to surgically unclog the blocked vessel(s), the use of recombinant-tissue plasminogen activator (rtPA) is another available therapeutic agent for clot dissolution. rtPA is the only currently approved drug for the treatment of ischemic stroke, but its use is limited by its short therapeutic time window of between 3 and 4.5 h [5].

Cellular therapy using stem cells—embryonic stem cells, and pluripotent stem cells, for the management of neurodegenerative diseases is attractive but limited by strict ethical requirements and tumorigenesis side-effects as reported in numerous animal studies [6–8]. In recent times, the use of mesenchymal stem/stromal cells (MSCs) is gaining a lot of attention as a potential neuroprotective and anti-stroke therapy. Here, we highlight the different cellular and molecular alterations that occur following an incidence of IS and how hypoxia and cytokine priming can be used to enhance the stroke-relevant physiological and pharmacological properties of MSCs.

## Ischaemic stroke and accompanying molecular and cellular changes

**Fig. 1:**
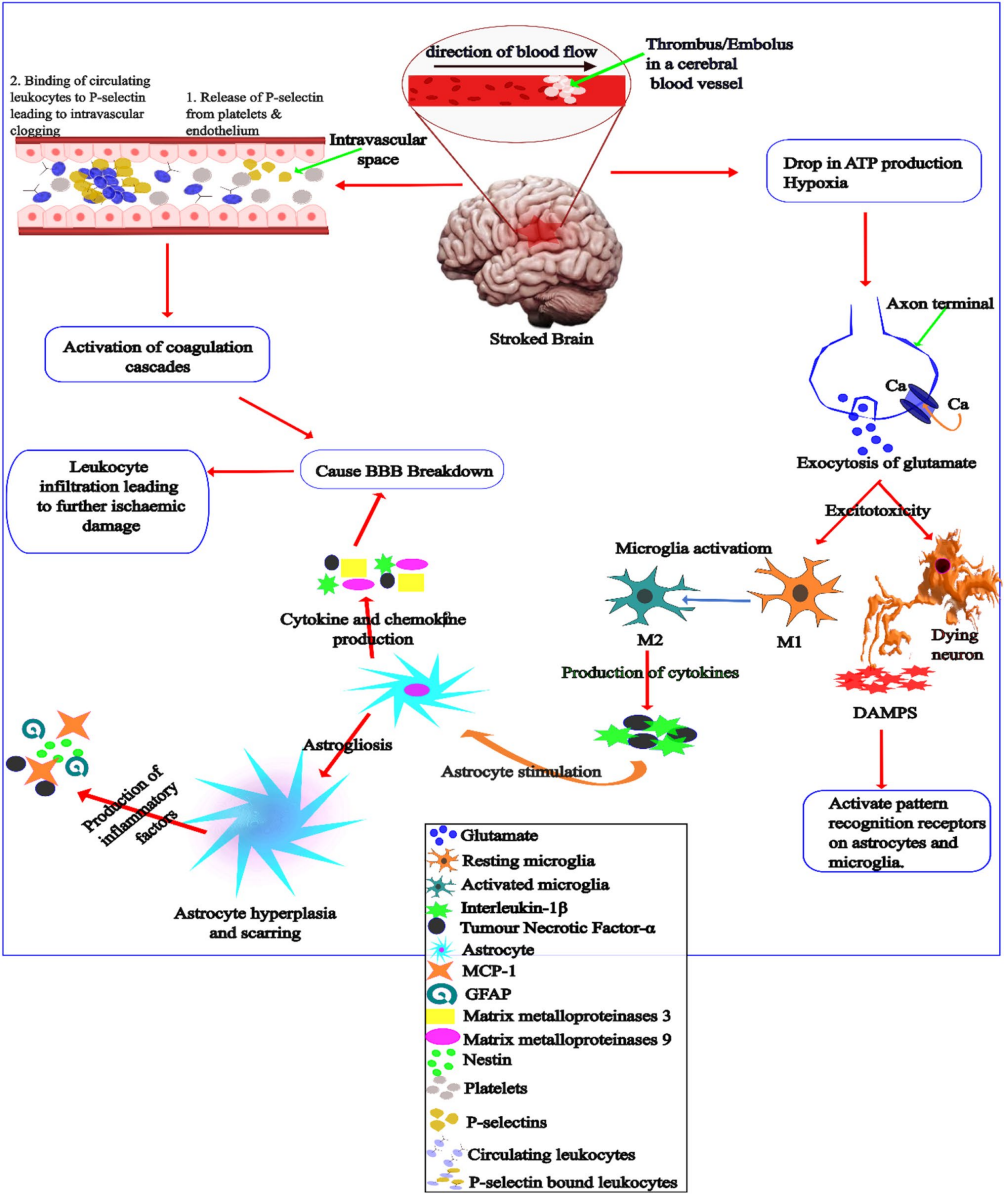
Molecular and cellular changes that occur following ischaemic stroke. Cerebral vascular occlusion results in oxygen and energy deprivation, followed by the formation of reactive oxygen species and accumulation of intracellular calcium. Influx of calcium leads to glutamate release from neuronal terminal, which causes excessive depolarization of neurons, hence excitotoxicity. Excitotoxicity causes microglia activation and the release of DAMPs from dying neurons culminating in a cascade of events including, inflammation and cerebral oedema

In the brain and under normal physiologic condition of sufficient oxygen, adenosine triphosphate (ATP), which is the main energy source of the CNS, is generated primarily in the mitochondria via oxidative phosphorylation. The obstruction to the flow of blood anywhere in the brain is followed by cellular, morphological, and molecular changes to different cells in the CNS, including neurons, glial cells, astrocytes, microglial cells, and blood vessels. Interruption of blood supply to the brain initiates a cascade of neuronal events culminating into neuroinflammation, oxidative stress, excitotoxicity, apoptosis, and autophagy. Generally, vascular occlusion results in deprivation of oxygen and energy, followed by the formation of reactive oxygen species, accumulation of intracellular calcium, release of glutamate, which lead in turn to later consequences, including cerebral oedema and inflammation, which can also contribute to brain damage. After a stroke incident, these changes occur as an attempt to mitigate the harmful effect of stroke and can sometimes also worsen the condition. In the initial time following stroke, the cells in the ischaemic penumbra (the regions surrounding the infarct core) activate signal pathways for survival, and these pathways can remain active for few hours to as long as many days [9]. See Fig. 1.

### Neuroinflammation

The innate immune system plays a critical role in both CNS physiology and pathology. Ischaemic stroke triggers a series of events collectively known as neuroinflammation, which is a complex process involving a tight interaction between the innate and adaptive immune system as well as an array of biochemicals including neurotransmitter (NT), chemokines, cytokines, and other factors. Intravascular inflammatory cascade activation begins immediately after a blood vessel is occluded even though immune response to CNS disruption manifest much later [10, 11]. Once there is a halt to blood flow, the altered flow of blood induces shear stress in the affected vessel which leads to the deployment of P-selectin, an adhesion molecule, to the cell surface, consequently causing leukocytes clogging within the affected blood vessels and contribute to ischaemic damage [11]. Other adhesion molecules such as ICAM-1, VCAM-1, and E-selectin, are also expressed following the activation of cytokines and endothelial pattern recognition receptors [12]. This is followed by the activation of the coagulation cascades which also worsens inflammatory cues. The inflammation within the affected blood vessels set the stage for BBB breakdown and infiltration of leukocytes. Inflammation in the brain neurons, initiated by CNS immune cells activation, begins almost immediately after intravascular inflammation has destroyed BBB integrity.

Within the brain parenchyma, astrocyte and microglia become activated some hours after ischemia and trigger a cascade of inflammatory events. In the acute phase of stroke this reaction is beneficial to minimize the degree of damage and limit the spread of infarct. However, as stroke progresses, the activities of the glial cells become detrimental leading to harmful brain damage that manifest as injury and/or death of neurons [13]. Activated microglia causes the production of tumour necrosis factor-alpha (TNF-α) and the release of IL-1β, which lead to the production of cytokines and chemokines by astrocytes and endothelial cells via a feedback mechanism on the inflammation cascade [13, 14]. Like macrophages during systemic inflammation, microglia phagocytose cellular debris and foreign organisms, and produce MMPs and cytokines capable of compromising the function of BBB [10]. The production of MMPs, cytokines and NADPH oxidase-mediated generation of ROS further exacerbate the neurotoxicity caused by activated microglia [10, 15].

Astrocytes, which are the most abundant of the glia cells also play a role in neuroinflammation following stroke. In a non-diseased state, astrocytes perform several functions including, contributing to the formation and protection of BBB, form glial limiting membrane barriers, provide plasticity and structural support to the CNS through their cytoskeletal network, replace damaged neurons via astrocytosis, and more. Astrocyte also take up excess glutamate from extracellular space into neurons where the neurotransmitter is converted to glutamine for reuse in NT synthesis [16]. However, this function is impaired during ischaemic stroke and a defect in the glutamate transporter during stroke has been suggested as the possible mechanism for such loss of function [17]. Following ischemia, neurons and other glia cells release cytokines, which causes reactive astrocyte proliferation. This hyperplasia induces the expression of various inflammatory factors such as GFAP, nestin, vimentin [18], IL-1β, and monocyte chemoattractant protein-1 (MCP-1) [19] leading to astrogliosis and scar formation. The stroke-induced failure of Na^+^/K^+^ pump causes astrocyte to swell leading to a rise in intracerebral pressure with a corresponding drop in cerebral perfusion [20]. Ischemia also induces the production of MMP9 which degrades the basal lamina, thus destroying the connection between astrocytes foot processes and the endothelial cells [21]. Consequently, leading to the rupture of the BBB that paves the way for the infiltration of peripheral inflammatory cells [22].

### Oxidative stress

The production of reactive oxygen species and other free radicals during stroke is a consequence of not only inflammation but also excitotoxicity and the inhibition of cellular respiration in a low oxygen environment [23]. These molecules, such as hydroxyl radical, superoxide, and peroxynitrite, are highly reactive and damaging to multiple cellular components, leading to cell death. Although nitric oxide is a normal signalling molecule in the body and has beneficial effects in stroke, larger amounts resulting from increased activity of the induced nitric oxide synthase (iNOS) can lead to aberrant signalling and or react with superoxide to produce peroxynitrite. Further damage can occur following reperfusion, because of the production of reactive oxygen species when oxygenation is restored. Reperfusion injury may be a vital component in stroke patients. The lesion produced by occlusion of a major cerebral artery consists of a central core in which the neurons quickly undergo irreversible necrosis, surrounded by a penumbra of compromised tissue in which inflammation and apoptotic cell death develop over a period of several hours.

### Excitotoxicity

Glutamate, the primary excitatory NT in the CNS plays a crucial role in neuronal cell death by its action on ionotropic N-methyl-d-Aspartate (NMDA) and (α-amino-3-hydroxy-5-methyl-4-isoxazolepropionic acid) (AMPA) receptors. Stroke causes depletion of neuronal oxygen and energy reserves leading to the release of toxic amounts of glutamate into the extracellular space [24, 25]. The glutamate released acts on NMDA receptors (NMDAR), both NMDA receptor 2A or 2B (NR2A or NR2B), to cause calcium accumulation. Inhibiting the influx of calcium into cells following glutamate receptor activation may also be beneficial in stroke. For instance, the overexpression of the transient receptor potential canonical 6 (TRPC6), a member of calcium conductive channel in the TRPC family, suppresses the increase in Ca^++^ induced by NMDA and reduces infarct size and mortality in mice [26]. Hyperforin, a lipophilic constituent in St. John’s Wort, is an activator of TRPC6 that has been found to be neuroprotective following transient middle cerebral artery occlusion (tMCAO) in rats [27]. In addition to NMDA, other glutamate receptor-independent non-selective cation channels also play significant role in ischaemic stroke [28]. Two of such receptors are the transient receptor potential melastatin (TRPM) and acid-sensing cation (ASCI) channels. TRPM2 and TRPM7 for instance have been reported to cause a delay in neuronal cell death following cerebral ischemia [29, 30]. Ginsenoside-Rd, a compound from Chinese Panax ginseng, exerts its neuroprotective effects in rats subjected to middle cerebral occlusion (MCAO) model of stroke by suppressing the expression of the calcium channel TRPM7 [28].

### Apoptosis

Apoptosis (programmed cell death) occur during stroke. The ATP depletion due to intravascular occlusion is followed by the loss of membrane potential and neuronal depolarization, leading to Ca^++^ influx and the release of glutamate. Binding of the released glutamate to NR2B receptor triggers apoptosis-associated excitotoxicity [31]. Taken together with excitotoxicity and oxidative stress, the energy depletion leads to the initiation of necrosis or apoptosis. Depending on the amount of ATP available in the affected brain region, cell death can be by either necrosis or apoptosis. Generally, neurons in the ischaemic core die by necrosis whereas those in the penumbra are lost to apoptosis [32]. Post-ischemia apoptosis occurs via both intrinsic (mitochondria apoptosis) and extrinsic (death receptor pathway) mechanisms. Mitochondria apoptosis occurs via either caspase independent or caspase dependent pathway. In the former, which take place within the nucleus, the activity of poly (ADP-ribose) polymerase (PARP) is stimulated by apoptosis-inducing factor (AIF) released from the mitochondria. In the latter, there is release of cytochrome C into the cytosol from the mitochondria and this activate caspase-3 from caspase-9, which triggers a cascade of caspase activity resulting in the destruction of cellular components, and ultimately, cell death [33]. Endogenous molecules known as caspase inhibitors of apoptosis (cIAPs) are present in the body to prevent caspase-dependent cell death by blocking the activation of caspases. Ischaemic preconditioning (IP) of neonatal rats was shown to confer protection against stroke induced by right carotid artery occlusion.

The extrinsic mechanism of apoptosis involves the activity of plasma membrane-bound death receptors, Fas and tumour necrotic factor receptor 1 (TNFR-1) [34]. During ischaemic stroke, the expression of genes such as the Fas ligand (FasL) is upregulated. To initiate apoptosis, FasL binds to Fas receptor leading to the recruitment of Fas-Associated death domain (FADD) proteins. FADD binds to procaspase-8 to form a complex known as death-inducing signalling complex (DISC) which catalyzes the generation of caspase-8 from procaspase-8 [35]. Caspase-8 triggers a cascade of events that lead to the production of caspase 3, then PARP, and ultimately, apoptosis [33].

### Autophagy

Cellular self-eating known as autophagy, is a normal physiological process that is carried out by lysosomes to maintain homeostasis in the face of metabolic stress or starvation. Three types of autophagy; chaperone-mediated autophagy, microautophagy and macroautophagy are recognized. Microautophagy is the most common form of autophagy and involves the sequestration of cytoplasmic content in a vacuole known as autophagosomes for onward delivery to lysosomes for degradation [36]. Accumulating evidence from several studies have revealed the activation of autophagy in many brain cells including neurons, macroglia, microglia, endothelial cells, and cerebral blood vessels during ischaemic stroke. Autophagy displays a paradoxical role in its response to cellular damage – absorbing damaged components as a protective measure in some cells and serving as a mechanism of cell death in others. Autophagy is mediated by the *Atg* family of proteins and regulated by several nutrient and energy sensing pathways including ULK1 kinase complex, that converge on the mammalian target of rapamycin (mTOR) [37–39].

Autophagy regulation in neurons can have significant implications for neuronal survival and recovery. Studies have shown that ischemic stroke can activate autophagy in neurons as a protective mechanism against cellular damage and protein aggregation [40, 41]. Autophagy helps in the clearance of damaged organelles, misfolded proteins, and other cellular debris, thereby promoting cell survival and preventing the build-up of toxic substances [42]. During the acute phase of ischaemic stroke, there is evidence of increased autophagic activity in neurons. This heightened autophagy response aids in the removal of damaged mitochondria (mitophagy) and the elimination of protein aggregates (aggrephagy) that can contribute to cell death [43, 44].

Furthermore, various signalling pathways and molecular regulators are involved in modulating autophagy in neurons after an ischemic event. Activation of the mammalian target of rapamycin (mTOR) pathway, for instance, inhibits autophagy, whilst inhibition of mTOR or activation of AMP-activated protein kinase (AMPK) typically promotes autophagy induction. Additionally, transcription factors such as hypoxia-inducible factor-1α (HIF-1α) and nuclear factor erythroid 2-related factor 2 (Nrf2) have been implicated in regulating autophagy during ischaemic stroke [44, 45]. These factors are responsive to oxygen and nutrient deprivation and can influence the expression of genes involved in autophagy pathways. The administration of chemicals such as 3-methyladenine (3-MA) have been used to demonstrate the role of autophagy in neuronal death. 3-MA is an inhibitor of phosphoinositide-3-kinase (PI3K) and a selective inhibitor of autophagy. When administered 30 min prior to the induction of ischaemia, it increased the expression of cleaved caspase 3 and aggravated neuronal apoptosis [46]. In contrast, post-ischaemic administration caused a significant reduction in infarct size and neuronal autophagy [47]. Understanding the precise mechanisms and dynamics of autophagy regulation in neurons following ischemic stroke is an area of active research. By deciphering these processes, future investigations will aim to identify potential therapeutic targets and interventions that can enhance autophagy-mediated neuroprotection and facilitate neuronal recovery after stroke.

Although evidence have shown that glial cells, unlike neurons, are more resistant to ischaemic stroke-induced autophagy [48], astrocytes and microglia nonetheless undergo autophagy. In 2016, Hu and colleagues reported an increased formation of autophagy vacuoles on astrocytes and overexpression of LC3-II and Beclin-1 (two markers of autophagy) following intracaudate thrombin administration [49]. Further, stroke induction in mice using MCAO or common carotid artery (CCA) occlusion resulted in extensive microglia activation and autophagy, and the administration of 3-MA attenuated this effect with a corresponding decrease in infarct size and neurological deficits [50, 51]. As with the case of neurons, autophagy regulation in glial cells following ischemic stroke also involves the mammalian target of rapamycin (mTOR) pathway and AMP-activated protein kinase (AMPK). The former acts as a negative regulator, inhibiting autophagy, whereas the activation of AMP-activated protein kinase (AMPK) promotes autophagy, thus playing a positive regulatory role. As part of the cellular adaptation to ischemic stress, HIF-1 also modulates autophagy in glial cells.

It is important to note that the field of autophagy regulation in the context of ischemic stroke is still evolving, and ongoing research continues to shed light on the complexities of these molecular pathways and their functional significance in neuronal survival and repair.

## Considerations for stroke therapy

As discussed above, ischaemic stroke is marked by inflammation, abundance of reactive radicals, low oxygen tension accompanied with other changes including, damage to cerebral blood vessels, injury/death of neurons, death of resident immune cells, microglia and supporting cells, astrocyte. An ideal stroke therapy should hence be able to favourably address and resolve most of these changes whilst in a condition of low oxygen tension and inflammation, as seen in ischaemic stroke. This therapy should promote the synthesis of new and viable blood vessels (angiogenesis), enhance the formation and maturation of new neurons (neurogenesis), resolve the neuroinflammation and neurotoxicity, cause a significant upregulation of relevant trophic and growth factors such as BDNF, TGF-β, and nerve growth factor (NGF), promote the drive of microglia to an anti-inflammatory phenotype, and remain in the ischaemic penumbra long enough for significant neural circuit reconstruction to occur. Stem cells immediately comes to mind as a potential therapeutic candidate with majority of these attributes. However, the use of totipotent and most pluripotent stem cells in regenerative medicine is limited by ethical concerns, and this has led to the emergence of numerous alternatives with similar properties, of which MSCs are most favoured.

## Mesenchymal stem/stromal cells (MSCs) in stroke research

MSCs are adult-derived stem cells that are capable of multilineage differentiation and self-renewal often administered/transplanted systemically or locally (directly to injury site) via various routes including intravenous, intraarterial, intrathecal, intramuscular, and intra-articular routes [52–56]. These cells are identified based on their physical properties, phenotype, and immunomodulation [57]. MSCs found popularity in regenerative medicine owing to their anti-inflammatory, self-renewal, immunogenic, immunomodulatory, tissue repair properties, and less stringent ethical concerns. These properties, with exception of the last, are the effect of a cocktail of bioactive chemicals, known as the secretome, that are released by MSCs into the microenvironment. The secretome contain bioactives such as cytokines, hormones, growth factors, chemokines, exosomes, microvesicles, lipid mediators, adhesion molecules, etc. [58], that play vital roles in cell-to-cell and paracrine communication, allowing MSCs perform their numerous biological roles. These biogenic molecules are also largely responsible for the numerous functions of MSCs such as cell differentiation and proliferation, immune signalling, angiogenesis, and intercellular interactions [59]. Additionally, MSCs are ubiquitous in the body with their physiological properties being dependent on their origin. Numerous experiments in animal stroke models have alluded to the potentials of MSCs as therapy for IS. In many of such experiments, MSCs were shown to improve behavioural outcome, increase functional recovery after stroke, reduce infarct volume, cerebral oedema and promote neural circuit reconstruction [60–67]. Despite these encouraging results, clinical trials using MSCs have only proven the relative safety of MSCs, but yet to show appreciable anti-stroke and neuroprotective potentials, albeit these human trials are still very few in number (NCT01019733, NCT04093336, NCT01297413, NCT03186456, NCT02580019, NCT04590118, NCT01310114, NCT04097652) [68–76]. These poor clinical outcomes can be attributed to the reduced efficacy of MSCs as a result of *inefficient migration and engraftment* and *tumorigenicity* (See Table 1).

**Table 1:** A description of different clinical trials involving the use of mesenchymal stem/stromal cells for ischemic stroke

Clinical Trials	Cellular therapy	Study Population	Route of Administration	Recorded/Anticipated Adverse Effects	Study Phase	Status
NCT01019733	Autologous Stem Cells	Children (1–8 years)	Intrathecal	Headache, vomiting, fever and stiff neck	Phase I	Completed (2011)
NCT04093336	Allogeneic human umbilical cord mesenchymal stem/stromal cells		Intravenous infusion	Tumorigenesis, death, pulmonary embolism, allergy, newly cerebrovascular events, and other adverse events	Phase I/II	Ongoing
NCT01297413	Allogeneic adult mesenchymal bone marrow stem cells	Adults (≥ 18 years)	Intravenous infusion	Urinary tract infection and intravenous site irritation	Phase I/II	Completed
NCT03186456	Allogeneic umbilical cord mesenchymal stem cells	Adults (40–75 Years)	Intravenous transplantation	Not yet known	Phase I	Ongoing
NCT02580019	Human umbilical cord mesenchymal stem cells	Adults (18–70 years)	Intravenous injection	Not yet known	Phase II	Recruiting
NCT04590118	Ischemia Tolerant Human Allogeneic Bone Marrow Mesenchymal stem Cells (it-MSCs)	Adults (≥ 18 years)	Intravenous injection	Not yet known	Phase I/IIa	Ongoing
NCT01310114	Human Placenta-Derived Cells PDA001	Adults (18–80 years)	Intravenous injection	Not published	Phase II	Terminated
NCT04097652	UMC119-06 (ex vivo cultured human umbilical cord tissue-derived mesenchymal stem cells)	Adults (20–80 years)	Intravenous injection	Not yet known	Phase I/II	Ongoing

Mesenchymal stem/stromal cells (MSCs) have made it to phase I and II clinical trials for the treatment of ischemic injuries including stroke, across wide age range—children, young adults, adults, and older adults. The phase I clinical trials are mostly aimed at ascertaining the safety of MSCs and their efficacy are the target of Phase II trials (also known as secondary outcome). The few concluded clinical trials reveal mild side effects as the main adverse events following MSCs transplantation.

*Inefficient migration and engraftment.* Generally, before administered MSCs can exert their beneficial effects, they need to migrate to the site of injury and engraft there to initiate and complete the process of repair and tissue regeneration. Homing of MSCs to injury site is regulated by a plethora of cytokines, chemokines, and growth factors. For instance, it has been revealed that the migration of bone-marrow-derived MSCs (BM-MSCs) to the site of injury is controlled by the chemokine CXC receptor 4 (CXCR4) under the influence of stromal derived factor 1 (SDF-1) [77]. Cytokines such as osteopontin also promote the migration of BM-MSC by upregulating the expression of integrin β1 [78, 79]. Growth factors such as transforming growth factor β1 (TGF-β1), vascular endothelial growth factors (VEGF), insulin-like growth factor 1(IGF-1), basic fibroblast growth factor (bFGF), platelet-derived growth factor (PDGF), and hepatocyte growth factor (HGF) are all involved in the MSCs-induced tissue regeneration as well as MSCs homing to injury site [80–85]. The ability of MSCs to migrate and home is, however, lost during in vitro cell culture [86], an occurrence that has been attributed to aging during cell culture [87, 88]. This is further confounded if the MSCs are sourced from aged donors due to altered composition and function of glycerophospholipids [89]. Accordingly, challenge of poor homing can be mitigated by sourcing MSCs from younger donors. However, it is unpracticable to use MSCs as soon as they are isolated. An in vitro expansion in culture media is necessary to produce a reasonable volume/concentration of MSCs to elicit therapeutic efficacy. Hence, a high number of culture passages is almost inevitable.

*Tumorigenicity.* Several published reports demonstrated the safety of MSCs, and several reports have also reported their tumorigenic effects [90–94]. As is observed in chronic non-/slow-healing wounds, MSCs are also recruited by tumour cells to support their growth and migration/metastases. Interestingly, most of the properties of MSCs that enable their homing have also been implicated in their tumorigenicity. Moreover, the immunosuppressive property of MSCs which is fostered via the secretion of soluble factors and mediators like prostaglandins E (PGE), indoleamine-2,3-dioxygenase (IDO), nitric oxide (NO) [95–97], etc., as a result of their interactions with natural killer cells, dendritic cells, macrophages, B and T cells, enable tumour cells to evade host immune surveillance, thus, enhancing MSC’s tumorigenicity. Further, the secretion of trophic factors, growth factors, proangiogenic factors (PDGF, TGFβ, VEGF, bFGF), cytokines (such as IL-6 and IL-8), and the differentiation of MSCs to endothelial-like cells or pericytes favours angiogenesis in tumour cells [93, 98, 99].

The question therefore arises as to how to harness the beneficial physiological properties of MSCs as efficacious stroke therapy while eliminating or minimizing these unwanted effects. One proven approach to achieve this is through MSC priming.

### Priming of MSCs with cytokine and hypoxia

**Fig. 2:**
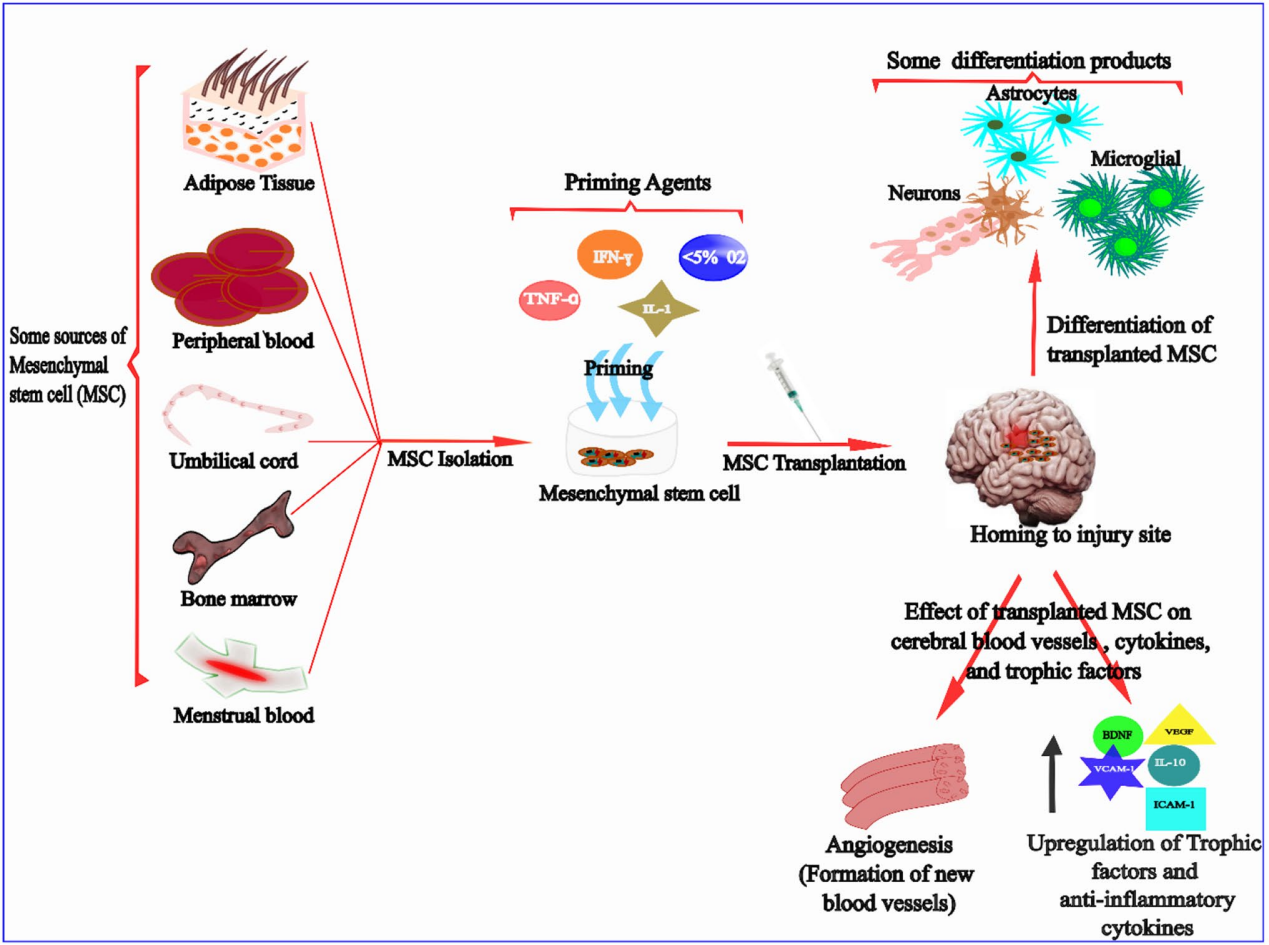
The effect of priming on different sources of mesenchymal stem/stromal cells (MSCs). Mesenchymal stem/stromal cells are isolated from various sources including bone-marrow, umbilical cord, menstrual blood, peripheral blood, and adipose tissue, via either an invasive or non-invasive method. The isolated MSCs are cultured using appropriate nutrient media and then conditioned with a single or a combination of priming agents such as IL-1, TNF-α, IFN-γ, and hypoxia. The primed MSCs are subsequently transplanted into an organism via local or systemic route. Within the organism, MSC migrate to the site of injury, a process known as homing. Here, MSCs induce the process of tissue repair and remodelling by differentiating into neurons, astrocytes, microglia and stimulating the development of new blood vessels and increase the expression of trophic factors, adhesion molecules like ICAM-1, and anti-inflammatory cytokines such as IL-10

In addition to its neuroectodermal differentiation (into neurons and glial cells) and self-renewal properties, an ideal MSC for IS therapy should be such that; (1) it can be isolated in ample amount from a viable and ethical source, and proliferate rapidly to ensure constant and continuous supply (2) have low immunogenicity to reduce the possibility of transplant rejection (3) should not be prone to rapid ageing to enable an efficient repair and remodelling (4) should promote angiogenesis, neurogenesis, and neural circuit reconstruction and (5) should not cause tumour formation nor promote the growth and metastasis of existing tumour. Because having an ideal MSC is not practicable, priming is done to make up for the deficiencies in any given MSC. Priming is the process of enhancing the biological and therapeutic features of MSCs. This process is also known as activation, preconditioning, or licensing. Generally, MSCs are licenced to redirect their secretome towards an anti-inflammatory phenotype [100], improve their engraftment following administration or transplantation, increase vascularization, and prepare them for their natural microenvironment [101–104]. In the context of ischaemic stroke – where is there is oxygen deprivation and inflammation, it is also desired that priming of MSCs ensures their survival under hypoxia without compromising their therapeutic effect. Priming of MSCs with proinflammatory cytokines and hypoxia offers such promising advantage. Although there is paucity of data on the effect of cytokine or hypoxia-primed MSCs in ischemic stroke, literature evidence notwithstanding provides convincing supports for stroke-relevant properties of cytokine and hypoxia priming of MSCs (Fig. 2).

#### Cytokine priming

The MSC secretome is hypoimmunogenic at a normal haemodynamic state due to the presence of low levels of class I major histocompatibility complex (MHC) and the absence of both class II MHC and co-stimulatory molecules [105, 106]. By implication, they undergo reduced proliferation, immunomodulation, and immunosuppression. To make MSCs suitable therapeutic agents in inflammatory conditions like IS, preconditioning them with proinflammatory cytokines (interleukins e.g., IL-1α, IL-1β, IL-1, TNF-α, and interferon-γ, IFN-γ) drive the secretome towards an anti-inflammatory phenotype [107, 108]. Used in small quantities, they increase MSCs’ therapeutic robustness by enhancing their immunosuppressive properties through upregulation of IDO and the increased secretion of factors like PGE2, HGF, adhesion molecules like intercellular adhesion molecule-1 (ICAM-1), and chemokine ligands (e.g., CXCL9, 10 and 11). Preclinical studies have confirmed this priming effect. For instance, IFN-ɣ was able to re-establish the immunosuppressive activity of senescent MSCs without upregulating HLA-DR expression [109]. In other studies where IFN-ɣ was used either alone or together with other proinflammatory cytokines, it was observed that such preconditioning caused an increased expression of IDO with accompanying increase in the levels of IL-10, as well as a decline in the levels of proinflammatory cytokines [110, 111]. TNF-α is reported to amplify the expression of immunomodulatory agents such as HGF, IDO, and PGE2 on MSC [112]. In combination with IFN-ɣ, TNF-α priming caused the differentiation of monocytes to IL-10-secreting macrophages which suppressed T-cells proliferation thereby enhancing the immunosuppressive property of MSC [110]. This cytokine also enhanced homing following intramuscular administration of the activated MSC and promoted tissue repair through the release of pro-angiogenic cytokines [113]. In 2017, Redondo-Castro and colleagues reported an increase in the production of the trophic factor granulocyte colony-stimulating factor (G-CSF) and anti-inflammatory cytokine IL-10 by BM-MSCs following IL-1 activation [114]. Similarly, IL-1β-priming of BM-MSCs activated the genes responsible for cell survival, migration, and adhesion. The authors also observed an increase in the production of chemokines, growth factors, MMPs (1 and 3) and adhesion molecule ICAM-1 [115]. Secretome from IL-1-primed MSCs were also able to significantly reduce infarct volume in MCAO model of stroke in mice [114, 116].

#### Hypoxia priming

Oxygen tension plays a vital role in cell fate, tissue function, and foetal development. Generally, oxygen tension begins to drop once it gets to the lungs and the decline continues with decreasing blood vessels supply. The variation in oxygen tension across different tissues depend largely on the tissue location and the level of vascularization—ranging from approximately 3% in the uterus following embryo implantation to 7% in the bone marrow and approximately 5% in a normal healthy brain [117–119]. On the contrary, MSCs are often cultured in the laboratory where oxygen tension is usually between 20 and 21%. Cell expansion in such a normoxic environment can induce stress in the cells and reduce their viability in vivo where they are expected to adapt to a hypoxic niche especially in diseases such as stroke that is associated with tissue hypoxia. Studies have shown that culturing MSCs in a normoxic condition leads to the early emergence of senescence, delayed doubling time, and DNA disruption [120–122]. However, in studies where MSCs were activated under reduced oxygen tension (between 1 and 5%), it was observed that hypoxia *improved cell proliferation, enhanced the secretion of bioactive* factors such as HGF, VEGF, etc. [123, 124], *prolonged survival time following transplantation, increased angiogenesis, and delayed MSC senescence* [125–127]. All these activities have been linked mainly to the expression of hypoxia-inducible factor -1α (HIF-1α) after hypoxia priming [125]. Further, studies have shown significant positive correlation between HIF-1α levels and expression of chemokine receptors CX3CR1, CXCR4, and CXCR7, which are known to play a major role in homing of transplanted MSCs [125]. Furthermore, hypoxia preconditioning has been reported to prevent apoptosis while increasing the expression of trophic factors [128]. Hypoxia priming also promotes MSC’s tissue repair via HIF-1α-mediated autophagy. There is, however, need for caution to avoid high autophagy which has been linked to cell death. In addition, hypoxia has been reported to direct microglia towards the pro-inflammatory M1 phenotype in rats [129].

## Conclusion

Regardless of MSC tissue source, it is important to prime them for optimum benefit. Used alone, neither cytokine nor hypoxia priming is sufficient to fully optimize the stroke-relevant therapeutic properties of MSCs. As such, we hypothesize that co-priming of MSCs with a cytokine and low oxygen tension have the potential to enhance the anti-stroke therapeutic properties of MSCs.

## Data Availability

Not applicable.
